# Supplementary material for: A UMLS-based spell checker for natural language processing in vaccine safety
Source: BMC Med Inform Decis Mak. 2007 Feb 12;7:3. doi: 10.1186/1472-6947-7-3 (PMC1805499; doi:10.1186/1472-6947-7-3)
Supplement: Additional file 13 — RAP application directory. Sets up directory for RAP (RDF application for PHP) source code [file 1472-6947-7-3-S13.gz › rap/doc/phpdoc/li_utility.html]

xml version="1.0" encoding="iso-8859-1"?


utility

Description
:   Class trees  
    Index of elements  
    Todo List

Classes
:   FindIterator
:   IterFind
:   Object
:   RDFUtil
:   StatementIterator

Files
:   FindIterator.php
:   IterFind.php
:   Object.php
:   RdfUtil.php
:   StatementIterator.php

phpDocumentor v 1.3.0RC3
